# A double-blind randomized clinical trial on the suggestive effect of anxiety management questionnaires in dental emergencies

**DOI:** 10.3389/fpsyg.2024.1333594

**Published:** 2024-02-12

**Authors:** Carla Irene Benz, Celina Wolanski, Martina Piefke, Guglielmo Campus, Andree Piwowarczyk, Thomas Gerhard Wolf

**Affiliations:** ^1^Department of Restorative, Preventive and Pediatric Dentistry, School of Dental Medicine, University of Bern, Bern, Switzerland; ^2^Department of Prosthodontics and Dental Technology, Faculty of Health, Witten/Herdecke University, Witten, Germany; ^3^Department of Psychology and Psychotherapy, Faculty of Health, Witten/Herdecke University, Witten, Germany; ^4^Department of Cariology, Saveetha Dental College and Hospitals, SIMATS, Chennai, India; ^5^Department of Periodontology and Operative Dentistry, University Medical Center of the Johannes Gutenberg University Mainz, Mainz, Germany

**Keywords:** CAQ, coping mechanisms, dental anxiety, HAF, questionnaire, RCT, suggestive effect

## Abstract

**Objective:**

Dental anxiety is widespread among both children and adults. To diagnose dental anxiety, standardized anxiety questionnaires are recommended. Based on the suggestive nature of the questionnaires, the study aimed to find out whether asking respondents about personal coping strategies before dental treatment influences their anxiety.

**Methods:**

This prospective, double-blind, randomized controlled clinical trial included a total of 158 patients of a university dental clinic on emergency service. The intervention group (*n* = 82) received the Coping with Anxiety Questionnaire (CAQ) and the control group (*n* = 76) the Hierarchical Anxiety Questionnaire (HAF). State anxiety scores were assessed by using the State–Trait Anxiety Inventory (STAI) before and after the completion of each questionnaire.

**Results:**

Anxiety decreased in the intervention group (CAQ) (*p <* 0.001) and increased in the control group (HAF) (*p <* 0.001).

**Conclusion:**

Within the limitations of the current study, a diagnostic tool of a standardized questionnaire for the assessment to assess personal coping strategies decreased state anxiety in comparison to a questionnaire assessing anxiety.

**Clinical trial registration:**

https://www.drks.de, German Trials Register (DRKS00032450).

## 1 Introduction

The level of anxiety associated with a dental visit is approximately 60–80% of the population (Rowe, 2005; Oostering et al., 2009; Sartory and Wannemüller, 2010). Patients aged 19–29 years have the highest levels of dental anxiety (Eitner et al., 2006), with a higher prevalence in women than in men (Hoefert and Jöhren, 2010). Dental anxiety can be triggered differently from person to person, including by the thought of visiting the dentist, the typical smell, or certain situations (Oostering et al., 2008). Although nowadays there is the possibility of almost painless treatment, about 10% avoid visiting the dentist altogether (Hoefert and Jöhren, 2010). A distinction is made between dental anxiety (DA) without disease value (DAnoDV) and dental anxiety with disease value (DAwithDV) (Enkling et al., 2019). The diagnosis of DAwithDV is made by specialists and psychologists (Enkling et al., 2019). DAwithDV is defined as a pathological fear of a dental treatment situation that is accompanied by avoidance behavior and appears excessive in the face of factual dangers (Enkling et al., 2019). According to ICD-10, DAwithDV is referred to as dental treatment phobia and is assigned to a specific (isolated) phobia F40.2. In contrast, patients with DAnoDV are characterized by the fact that their anxiety is relieved by the exchange of information, education, and anesthesia (Enkling et al., 2019).

The assessment of a patient’s anxiety is usually based solely on the dentist’s experience. However, the available literature emphasizes that validated survey instruments should be used for this purpose, as is the case in psychology and other medical disciplines (Nebel et al., 1989). It has already been established in clinical studies that anxiety diagnostics seem to have a positive relevance (Kent, 1986; Humphris et al., 2006; Humphris and Hull, 2007). The patient’s anxiety was significantly lower when the dentist was aware of the anxiety (Dailey et al., 2002). An anxiety survey before dental treatment not only seems to have a positive effect on the doctor-patient relationship but also a positive influence on compliance and treatment success (Mercer and Reynolds, 2002; Neumann et al., 2009; Sangappa, 2012; Decety, 2020). Information collection can be done in a variety of ways. In addition to working with validated data collection instruments such as questionnaires, direct interviews with open or closed question format, as well as open narration of events.

In dental practice, the use of validated anxiety questionnaires has become established. Using the paper-pencil method, the available questionnaires are completed by the patients themselves. The most used anxiety questionnaires include the Dental Anxiety Scale (Corah, 1969), the Dental Fear Survey (Kleinknecht and Klepac, 1973), the Dental Cognitions Questionnaire (Milgrom et al., 1995), and the Hierarchical Anxiety Questionnaire (HAF) to generate information about anxiety-provoking stimuli (Jöhren, 1999).

However, although the benefits of anxiety questionnaires could be shown, information about the influence of these questionnaires is still largely unknown. Processing an anxiety questionnaire immediately before dental treatment is particularly challenging for anxious patients. The reason is that if a person is in an extraordinarily stressful or distressing situation like a dental treatment, the person is particularly susceptible to suggestion (Polczyk and Pasek, 2006). However, an individual level of suggestibility is common to all interviewing options (Kubinger and Jäger, 2003). Here, questioning by an interviewer can lead to the greatest measurable distortions of perception (Reutermann, 2006). The term suggestion describes a kind of influencing of humans, which always happens unconsciously (Kubinger and Jäger, 2003), and can be distinguished from conformity, compliance, lying, and error (Reutermann, 2006). Suggestions are evoked predominantly in connection with active communication and can lead to distortions of cognition. In this context, the adoption of suggestions is not modified by rational deliberation or reflex mechanisms and is adopted by the receiving person (Kubinger and Jäger, 2003). The detailed mechanisms of processing have not been conclusively researched. However, it can be stated that everyone is affected by suggestibility, only the degree of expression can be influenced by various individual factors. For example, anxiety leads to an increase in suggestibility (Dorsch et al., 1994). The field of suggestion research predominantly refers to direct questioning of individuals (Reutermann, 2006). Here, susceptibility to suggestion is viewed as a deficiency situation in which individuals draw on and internalize affective, cognitive, or structural aspects. Additionally, a lack of certainty or confidence plays a role (Kubinger and Jäger, 2003). Suggestions can be processed into specific information through indirect suggestions. This, in turn, induces stereotypes, and desired specifications, conclusions, and decisions can be suggested (Kubinger and Jäger, 2003). Thus, specific answers can also be suggested by specifically posed questions. Therefore, when evaluating a survey, it is no longer possible to determine whether the respondent’s answers are his or her own or were predetermined by specific questions (Köhnken, 1999). The manner of emotions, memory content, and exact circumstances recorded play a major role in the evaluation of responses. Ideally, any elicitation of memory content, emotion, and cognition should be done without disclosure, if possible. Accordingly, the questioning of subjects can be actively or passively guided. The more detailed the respondent is guided to an answer by the items asked, the greater the influence on the information collected. The subjects of many surveys are fear-inducing stimuli, e.g., in fear questionnaires. The latter can influence emotions by asking specific items and thus also influence the expression of the anxiety elicited in the questionnaire (Sporer and Bursch, 1997; Polczyk and Pasek, 2006; Hünefeldt et al., 2009; Nicolas et al., 2011).

Regarding the findings of suggestibility research - the suggestible character underlying all questionnaires and the increased susceptibility to suggestion in stressful or anxious situations - the current study aimed to find out whether asking respondents about personal coping strategies before dental treatment influences their anxiety. A commonly used questionnaire for dental treatment anxiety Hierarchical Anxiety Questionnaire (HAF) served as a control.

This investigation is based on the research hypothesis questioning if personal coping strategies have a fear-mitigating effect. Therefore, a double-blind, randomized controlled clinical trial was planned to investigate the influence of personal coping strategies questionnaires directly before dental emergency treatment.

## 2 Materials and methods

### 2.1 Study design and procedure

The present research project was planned as a prospective, randomized, controlled, clinical questionnaire study. After the positive ethical vote (No. 151/2020) by the ethics committee of the University of Witten/Herdecke, the patients of the dental emergency service of the University of Witten/Herdecke were acquired for participation. The study was registered retrospectively in the German Clinical Trials Register (DRKS-ID: DRKS00032450). The studies were carried out using an intervention group using the Coping with Anxiety Questionnaire (CAQ) and a control group using the Hierarchical Anxiety Questionnaire (HAF). The intervention group received the Coping with Anxiety Questionnaire (CAQ) (Schulz et al., 2010). This questionnaire has been used in the dental context (Lederer, 2010). The control group received the Hierarchical Anxiety Questionnaire (HAF) (Jöhren, 1999), an instrument for measuring anxiety in the dental context that has been validated several times in clinical studies in the German language (Coultman-Smith, 2008; Julian, 2011).

**Figure 1:**
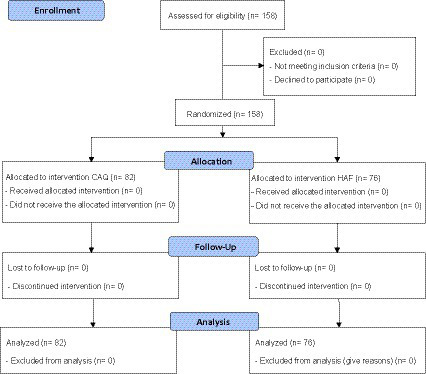
CONSORT 2010 flow diagram.

The order of study participation was randomized to the intervention or control groups according to the appearance of the patients at the dental clinics using a single sequence of random assignment. The participants chose an envelope without any indication. In the envelope, they found one version of CAQ (Schulz et al., 2010) or HAF (Jöhren, 1999) and twice the State–Trait Anxiety Inventory (STAI) (Lederer, 2010; Julian, 2011), whereby only the state version was used in the present study. The patients had to complete the three questionnaires before and after completion of the CAQ/HAF (order: STAI 1, CAQ/HAF, STAI 2) and before the dental emergency treatment. Another envelope without indication was given to both the patient and the dentist after the dental emergency treatment. This envelope contained a previously developed and validated questionnaire to assess anxiety and the questionnaires used. The questionnaires for the patient and the dentist after the dental emergency treatment were different. The data from these respective dental anxiety questionnaires will be published in a separate article. The dentists were informed using the cover story and blinded to the fact that the patients could only be called for treatment after completion of the questionnaire. The completed questionnaire and the respective results were given to the dental practitioner. Both the dentist and the patient were blinded to group assignment and were informed of the entire study (and cover story) at the end of the study because of possible bias, as multiple participation could not be ruled out at the time of the study. The detailed flowchart is shown in Figure 1 and the CONSORT 2010 flow diagram in Supplementary materials (Begg et al., 1996; Schulz et al., 2010).

### 2.2 Cover story and informed consent

Psychological questionnaire studies regularly use cover stories to initially disguise the true background of a study. In this way, the response tendencies of the subjects to social desirability can be circumvented (Bizo and Sweeney, 2005; Coultman-Smith, 2008). Consent to participate could be withdrawn at any time without giving reasons. In the current study, both the patient and the dental practitioner were informed about the study using a cover story. However, the cover story did not correspond to the actual research project because of the potential bias. Patients and dentists were informed that the University of Witten/Herdecke collects subjective data as part of the quality management process to make patients’ stay at the dental clinic as pleasant as possible. After completion of the questionnaires, patients and dental practitioners were informed about the cover story and the true background of the study. Patients signed a written informed consent form before and after the cover story.

### 2.3 Study sample, setting, and statistical analysis

A total of 158 subjects from a convenience sample of the dental emergency service of the University of Witten/Herdecke were acquired for the study over a period from December 1, 2020, to February 28, 2021. A sample size calculation was performed before the start of the study using the web-based platform OpenEpi (Sullivan et al., 2009), assuming a 5% difference between the two groups (Dailey et al., 2002; Humphris et al., 2006; Humphris and Hull, 2007). The bilateral significance level was set at 95% with a power of 90%. The number of patients to be included per group was set at 64. The sample size was increased by 20% to safeguard the estimates against a possible number of patients not wishing to participate in the study or subsequently withdrawing their written informed consent after the completion of the study. The following inclusion criteria were: adults with a minimum age of 18 years; subjects of European origin to reduce response biases; and with good level of written and spoken German to avoid language-related bias. The exclusion criteria were serious medical illness, serious mental illness, depression, bipolar disorder, neurological/psychiatric illness up to 2 years ago; and taking medication affecting the central nervous system. Subjects were subsequently excluded if dental treatment was not possible due to other factors, such as referral for further treatment to oral and maxillofacial surgery.

All questionnaires were completed using the paper-pencil method. Subsequently, the collected data were transferred to Excel® (Microsoft Corporation, Redmond, Washington, USA) and then checked for any mistakes and transferred to the statistical software STATA 17® for analysis. The difference between the two groups was evaluated before the start of the trial using when appropriate the analysis of variance oneway, Pearson’s chi-square test, and the Fisher Exact test (Razali and Wah, 2011). To verify differences between the groups, an analysis of covariance (ANCOVA) was carried out using The State–Trait Anxiety Inventory, which was collected during the second questionnaire round (STAI 2), as the dependent variable and STAI 1 as the covariate. The homogeneity of variances was determined by Levene’s test. In the current study, only the change in state anxiety was evaluated among the groups. The probability of error was set at 0.05 so that a hypothesis could be accepted or rejected with a probability of at least 95% (*p* = 0.05).

## 3 Results

The demographic characteristics of the convenience sample are shown by subjects in the intervention group (CAQ) and the control group (HAF) in Table 1. No statistically significant differences were observed among the two groups (Table 1), even if a higher percentage of women is present in the control group (HAF), 56.10% versus 52.63% in the intervention group (CAQ), resulting in a homogeneous subject distribution within the groups.

**Table 1:** Distribution and intergroup comparison of demographic sample description.

	Intervention group (CAQ) (*n* = 82)	Control group (HAF) (*n* = 76)
Age in years		
Mean (range)	44.58 (18–98 yy)	49.10 (18–81 yy)
ANOVA one-way *F* = 2.93 *p* = 0.09		
Gender		
Males	36	36
Females	40	46
Pearson’s *χ*^2^_(1)_ = 0.19 *p* = 0.66		
Marital status		
Single	30	33
Married	39	47
Widowed	6	2
Fisher Exact probability test *p* = 0.31		
Working		
Yes	54	54
No	20	17
Pearson’s *χ*^2^_(1)_ = 0.18 *p* = 0.67		
Insurance status		
Private	15	17
Statutory	60	65
Pearson’s *χ*^2^_(1)_ = 0.01 *p* = 0.91		
Dental visit		
Yes	50	51
No	25	31
Pearson’s *χ*^2^_(1)_ = 0.34 *p* = 0.56		

The state anxiety difference measured at the first round was statistically significant between the two groups 50.18 in the CAQ and 46.28 in the HAF group (*F* = 1.61 *p* = 0.02), the same feature was also observed when STAI 2 was compared (*F* = 1.63 p = 0.02). The intra-group comparison between the two measurements was also statistically significant both for the CAQ and HAF groups (Table 2). Preliminary analysis underlines that STAI 2 was statistically significantly different among the two sexes (STAI 2; males: mean 47.12 and standard deviation (SD) 12.58, versus females: mean 50.78 and SD 11.74, *F* = 3.65, *p* < 0.05) (*data not in tables*). The ANCOVA model with STAI 2 as the dependent variable showed a statistically significant difference between the groups (*p* < 0.001) (Table 3).

**Table 2:** The State–Trait Anxiety Inventory was measured in the first round (STAI 1) and (STAI 2) after the second round of the questionnaire.

Group	STAI 1 mean (SD)	STAI 2 mean (SD)	*F* value	value of *p*
Intervention group (CAQ) (*n* = 82)	50.18 (12.94)	46.54 (10.90)	3.16	<0.01
Control group (HAF) (*n* = 76)	46.28 (10.17)	51.89 (13.01)	3.14	<0.01
One-way ANOVA	*F* = 1.61 *p* = 0.02	*F* = 1.63 *p* = 0.02		

The differences were evaluated using a one-way analysis of variance (one-way ANOVA).

**Table 3:** Analysis of the covariance, STAI 2 was the dependent variable.

Covariates	Partial SS	df	MS	*F* value	value of *p*
STAI 1	12172.72	45	270.50	3.10	<0.01
Group	903.47	1	903.47	10.34	<0.01
Model	13883.00	47	295.40	3.38	<0.01
Residual	9611.952	110	87.38		
Total	23495.95	157	149.66		

Number of observations = 158 R-squared = 0.59 Root MSE = 9.35 Adj R-squared = 0.42.

## 4 Discussion

The aim of the current study was whether asking patients about their personal coping strategies before dental treatment shows an anxiety-influencing effect. The results of the present study show a statistically relevant reduction in anxiety using the CAQ as well as an increase in anxiety by processing the HAF, a frequently used German anxiety questionnaire in the dental setting.

Asking patients about their fear of dental treatment is of enormous importance before a dental procedure (Sartory and Wannemüller, 2010) although a benefit of anxiety questioning has previously been shown to be positive for the doctor-patient relationship and for treatment success (Mercer and Reynolds, 2002; Neumann et al., 2009; Sangappa, 2012; Decety, 2020). The influence of such questioning should be further investigated and critically evaluated regarding the actual benefit. Especially after the unexpected results of this study which showed that confrontation with a questionnaire asking about anxiety-provoking stimuli before dental treatment can increase patient’s anxiety. While it stands to reason that this increase in anxiety may result from the suggestible nature of anxiety questionnaires, i.e., HAF, the observed and statistically significant effect should be viewed critically. Even if there is a statistically significance difference between the STAI and the magnitude of the *F*-value and the value of *p* are similar, it is important to note that in one group a decrease (CAQ) and the other group (HAF) an increase in the state anxiety could be observed. A visit to the dental emergency service with an acute condition is an exceptional situation in a patient’s life. Even regular visits to the dentist are often associated with anxiety (Sartory and Wannemüller, 2010; Halsband and Wolf, 2015). Emergency dental treatment is often associated with anxiety-inducing stimuli such as the sound of dental drills or injections with local anesthesia (Halsband and Wolf, 2015), whereby the most common cause of toothache worldwide is dental caries that may result in root canal treatment, abscess incision or even tooth extraction (Douglass and Douglass, 2003; GBD 2017 Oral Disorder Collaborators, 2020). Failure to treat dental infections can lead to life-threatening infections (Douglass and Douglass, 2003; GBD 2017 Oral Disorder Collaborators, 2020; Hammel and Fischel, 2019). Increased anxiety may lead to a lack of compliance or even refusal of dental treatment, i.e., the patient may only be able to be treated using sedation or even general anesthesia. Such treatments, which are performed by an anesthesiologist, can pose enormous health risks, or lead to complications and economic burdens for the patient in the case of existing illnesses or allergies. An improvement in the subjective experience of dental treatment is highly relevant to prevent avoidance behavior before treatment and the resulting lack or non-provision of dental care (Halsband and Wolf, 2015). Questionnaires that can influence anxiety are therefore very important before dental treatment.

The Dental Anxiety Scale by Corah (1969) is one of the most widely used dental anxiety questionnaires worldwide (Corah, 1969; Newton and Buck, 2000). Numerous modifications have been developed for questioning in the dental context to accommodate the tight time management in the dental office; the most popular modification is the Modified Dental Anxiety Scale (MDAS) (Humphris et al., 1995, 2006). The validity and range of use of the MDAS are diverse and have been frequently tested (Humphris et al., 2009). However, questionnaire studies on dental anxiety overall indicate no negative influence of the questionnaires used on individual anxiety (Kent, 1986; Humphris et al., 2006), but highlight the positive benefits as a confidence-building measure between the patient and the dentist. Previous studies methodologically disregard the suggestibility of the questionnaire. In the present study, the STAI-S served as a measurement tool of anxiety before and after the completion of the anxiety or coping questionnaires. Such use of the measurement instruments has not occurred in previous studies, including placebo-controlled studies (Humphris et al., 2006; Humphris and Hull, 2007). In German-speaking countries, in addition to the DAS, the HAF is particularly widespread in the anamnesis of anxiety (Weifenbach et al., 2019). For this questionnaire, however, no comparable studies are yet available, as the working group around Humphris has done for the MDAS (Dailey et al., 2002; Oostering et al., 2008; Enkling et al., 2019). However, the difference between the DAS, MDAS, and HAF is crucial in terms of possible influence. Compared to Humphris’ studies (Dailey et al., 2002; Oostering et al., 2008; Enkling et al., 2019), the increase in anxiety, besides the study design, can be explained by the greater number of anxiety-inducing stimuli queried. In the present study, six additional stimuli were elicited (11 in total). The latter included the “typical smell of the treatment room,” “the position on the dental chair,” “looking at radiographs,” “the possible treatment of caries,” “the possible removal of a tooth,” and “picking up a scalpel” (Jöhren, 1999). The familiar anxiety-provoking stimuli, such as the naming of the instruments and especially the clear reference to the syringe with the words “the dentist changes the position of the chair and prepares a syringe” (Oostering et al., 2008), are explicitly recalled before the treatment. Further items deal with the drill: “Imagine you hear the typical sound of the drill - how do you feel?.” In addition, the topic of wisdom tooth extraction is also addressed: “A wisdom tooth is to be removed from you, the injection has already been placed. The dentist picks up the scalpel.” Since a visit to the dentist is often fraught with anxiety (Hoefert and Jöhren, 2010; Sartory and Wannemüller, 2010), and based on the underlying literature, it can be assumed that anxiety can increase the suggestibility of individuals (Ridley and Clifford, 2004). The question that arises is whether questioning using the HAF by suggestion leads to an increase in anxiety levels. The basic prerequisite for successful suggestion in this context is the unconscious processing of the stimuli (Kubinger and Jäger, 2003), which occurs here using the HAF. Moreover, suggestions can only take place in the context of an interaction. In this case, the completion of the questionnaire takes over the interaction. Here, the type of interaction even plays a subordinate role (Gudjonsson and Clarke, 1986). As already demonstrated (Kubinger and Jäger, 2003), the suggestions within questionnaires can also influence the statements and feelings of the subjects in the present study.

To the best of the authors’ knowledge, this is the first study to examine the interrogation of coping strategies in the dental context. Watts created the “Coping with Anxiety” questionnaire in 1989 (Watts, 1989) and argued that, while directing attention to fear signals is an important step for processing, it aggravates the fear, or panic attack itself (Watts, 1989). After the questionnaire was modified several times, König’s German-language version was used (König and Hiebler, 2008). The reference by Watts himself that directing attention to fear signals is an important step for processing underpins the recommendation of a current guideline on dental anxiety (Enkling et al., 2019). The use of appropriate questionnaires such as the DAS, MDAS, or HAF is appropriate for the psychotherapeutic context. However, the results of this study indicate that the use of an anxiety questionnaire in dental practice can also be detrimental.

### 4.1 Limitations of the study

To verify the results collected in this study, they should be investigated or replicated in different study designs to confirm or refute the present results. Future studies should consider the purpose for which patients complete a questionnaire before treatment. However, several limitations should be considered. If dental treatment is imminent, the goal should be to guide the patient through the treatment as empathetically and safely as possible. In the best case, the patient then even experiences self-efficacy and can learn to get through dental treatment. Anxiety questionnaires could, if necessary, only be useful at an initial examination and consultation appointment and not immediately before emergency treatment. In particular, the duplicate survey of the STAI was met with incomprehension by many patients. Repeated testimony on identical items was problematic for some patients. Therefore, it should be considered whether the time interval between the collection of the STAI should be extended in the future. The higher percentage of women in the control group might have biased the results even if the two groups were not statistically significantly different regarding age and sex. To account for the natural fluctuation of anxiety in a dental emergency and to better distinguish the effects of completing the respective questionnaire, the use of an additional control group with a neutral subject in a future study could be beneficial in addition to the two frequently used questionnaires CAQ and HAF. Another limitation is the relatively small sample size, which is small compared to previous studies of similar questions.

## 5 Conclusion

Within the limitations of this study, it can be concluded that in emergency dental treatment a diagnostic tool of a standardized questionnaire for the assessment to assess personal coping strategies (Coping with Anxiety Questionnaire, CAQ) decreased state anxiety in comparison to a questionnaire assessing anxiety (Hierarchical Anxiety Questionnaire, HAF) that increased state anxiety in patients. Further prospective, longitudinal, multicenter studies with larger sample sizes should be conducted to verify the observed effects.

## Data availability statement

The raw data supporting the conclusions of this article will be made available by the authors, without undue reservation.

## Ethics statement

The studies involving humans were approved by ethics committee of the Witten/Herdecke University (Germany). The studies were conducted in accordance with the local legislation and institutional requirements. The participants provided their written informed consent to participate in this study.

## Author contributions

CB: Conceptualization, Data curation, Project administration, Writing – original draft, Writing – review & editing, Formal analysis, Funding acquisition, Investigation, Methodology, Software, Validation. CW: Conceptualization, Data curation, Funding acquisition, Methodology, Project administration, Software, Writing – original draft. MP: Conceptualization, Formal analysis, Writing – review & editing. GC: Data curation, Formal analysis, Writing – review & editing. AP: Conceptualization, Project administration, Writing – review & editing. TW: Conceptualization, Data curation, Formal analysis, Funding acquisition, Project administration, Writing – original draft, Writing – review & editing.
